# miR-188-5p inhibits tumour growth and metastasis in prostate cancer by repressing LAPTM4B expression

**DOI:** 10.18632/oncotarget.3341

**Published:** 2015-01-21

**Authors:** Hongtuan Zhang, Shiyong Qi, Tao Zhang, Andi Wang, Ranlu Liu, Jia Guo, Yuzhuo Wang, Yong Xu

**Affiliations:** ^1^ Department of Urology, National Key Specialty of Urology, Second Hospital of Tianjin Medical University, Tianjin Key Institute of Urology, Tianjin Medical University, Tianjin, China; ^2^ Vancouver Prostate Centre & Department of Urologic Sciences, Faculty of Medicine, University of British Columbia, Vancouver, British Columbia, Canada; ^3^ Department of Experimental Therapeutics, British Columbia Cancer Agency, Vancouver, British Columbia, Canada

**Keywords:** miRNA, metastasis, miR-188-5p, prostate cancer, LAPTM4B

## Abstract

Elucidation of the molecular targets and pathways regulated by the tumour-suppressive miRNAs can shed light on the oncogenic and metastatic processes in prostate cancer (PCa). Using miRNA profiling analysis, we find that miR-188-5p was significantly down-regulated in metastatic PCa. Down-regulation of miR-188-5p is an independent prognostic factor for poor overall and biochemical recurrence-free survival. Restoration of miR-188-5p in PCa cells (PC-3 and LNCaP) significantly suppresses proliferation, migration and invasion *in vitro* and inhibits tumour growth and metastasis *in vivo*. We also find overexpression of miR-188-5p in PC-3 cells can significantly enhance the cells' chemosensitivity to adriamycin. LAPTM4B is subsequently identified as a direct target of miR-188-5p in PCa, and is found to be significantly over-expressed in PCa. Knockdown of LAPTM4B phenotypically copies miR-188-5p-induced phenotypes, whereas ectopic expression of LAPTM4B reverses the effects of miR-188-5p. We also find that restoration of miR-188-5p can inhibit the PI3K/AKT signaling pathway via the suppression of LAPTM4B. Taken together, this is the first report unveils that miR-188-5p acts as a tumour suppressor in PCa and may therefore serve as a useful therapeutic target for the development of new anticancer therapy.

## INTRODUCTION

Prostate cancer (PCa) is one of the most common cancers among men and the second most common cause of male cancer-related deaths [1]. While multiple molecular events characterize PCa initiation, growth, invasion and metastasis, a detailed knowledge of the molecular mechanisms underlying its development and progression remains elusive. Despite the availability of an earlier diagnosis using serum prostate-specific antigen (PSA) and improved treatments, many PCa patients subsequently die following disease progression. PCa patients are generally androgen-sensitive at the initial diagnosis. However, patients eventually develop androgen-independent PCa. Chemotherapy is a choice for men diagnosed with androgen-independent PCa. Drug resistance poses a great challenge in treating chemorefractory PCa patients. Therefore, understanding the molecular mechanisms underlying PCa development and progression would help to improve therapies for the disease.

It's well known that microRNAs (miRNAs) contribute to the initiation, development and metastasis of various types of cancers [2]. Various types of cancers show aberrant expression of miRNAs that can function as either tumour suppressors or oncogenes. miRNAs are highly conserved endogenous small 20-25 nucleotide non-coding RNAs [3,4]. More than 50% of the known miRNAs have been shown to participate in tumorigenesis and/or metastasis by directly targeting oncogenes or tumour suppressors [5]. In PCa, an increasing number of studies have focused on the effects of miRNAs on PCa growth and metastasis. One miRNA may be able to target several pathways, facilitating cancer cells evasion of drug treatment and generating stem-like cells [6]. Therefore, it is of value to illuminate whether dysregulation of these miRNAs-regulatory networks are responsible for chemoresistance. Elucidation of the molecular targets regulated by the tumor-suppressive miRNAs can provide new insights into the mechanisms of PCa oncogenesis and metastasis and may facilitate the development of novel therapeutic strategies for the treatment of PCa.

To the best of our knowledge, the molecular mechanism of miR-188-5p deregulation and its regulatory networks in PCa remain elusive, so the present study aimed to clarify the biological functions and underlying molecular mechanisms of miR-188-5p in PCa. The data showed that miR-188-5p expression was significantly downregulated and associated with tumour metastasis and poor prognosis in PCa. We also demonstrated that the miR-188-5p/LAPTM4B/PI3K/AKT regulatory network played an important role in PCa progression and chemotherapeutic drug sensitivity.

## RESULTS

### miR-188-5p as a potential metastasis-associated miRNA

Using our miRNA array data from paired metastatic LTL-313H and non-metastatic LTL-313B PCa xenografts [7], a total of 55 downregulated and 49 upregulated miRNAs in the metastatic line were identified. Of the downregulated miRNAs, miR-188-5p showed a 10.46-fold decrease. We also conducted clinical PCa miRNA microarray data analyses and found that miR-188-5p is under-expressed in bone metastatic samples compared with primary PCa samples (Supplementary Figure 1) [8].

### Downregulation of miR-188-5p is associated with metastasis and poor prognosis in PCa

The correlation between miR-188-5p expression and clinicopathological characteristics was assessed in 180 PCa patients (Supplementary Table 1). The miR-188-5p expression level was categorized as low or high in relation to the median value. The results indicated the downregulation of miR-188-5p was significantly correlated with lymph node metastasis, stage, preoperative PSA, Gleason score, and biochemical recurrence, seminal vesicle invasion, and angiolymphatic invasion (Supplementary Table 1). In order to evaluate the predictive significance of miR-188-5p downexpression for lymph node metastases, univariate and multivariate logistic regressions were performed. We found that downregulation of miR-188-5p was independently associated with lymph node metastases (Supplementary Table 2). miR-188-5p high expression had a biochemical recurrence-free survival benefit compared with low expression, indicating that downregulation of miR-188-5p might be an important molecular mechanism in identifying outcomes for PCa patients. To study the impact of miR-188-5p downregulation on prognosis in PCa patients, the prognostic significance of miR-188-5p downregulation for biochemical recurrence-free survival and overall survival was investigated. Univariate Cox analysis showed that downregulation of miR-188-5p may affect both the biochemical recurrence-free survival and overall survival of PCa patients. Further adjustment of covariate factors by using multivariate Cox regression analysis confirmed that downregulation of miR-188-5p in PCa was an independent prognostic factor for poor overall survival and biochemical recurrence-free survival (Supplementary Tables 3 and 4). Collectively, these results suggested that downregulation of miR-188-5p was associated with metastasis and poor prognosis in PCa.

### miR-188-5p inhibits the proliferation, migration and invasion of PC-3 and LNCaP cell lines

To evaluate the biological functions of miR-188-5p during the progression of PCa, we re-introduced miR-188-5p into PC-3 and LNCaP cells. As shown by the cell proliferation and colony formation, we observed that ectopic miR-188-5p expression significantly inhibited cell proliferation and colony formation compared to control cells (Supplementary Figure 2A, B, C, D). We next determined whether miR-188-5p could affect migration and invasion of PCa PC-3 and LNCaP cells. The data showed that ectopic miR-188-5p expression significantly reduced the invasion and migration of PC-3 and LNCaP cells (Supplementary Figure 2E, F, G, H). Taken together, these observations demonstrated the downregulation of miR-188-5p can promote PCa progression *in vitro* by enhancing proliferation, invasion and migration.

### LAPTM4B as a direct target of miR-188-5p in PC-3 and LNCaP cells

**Figure 1:**
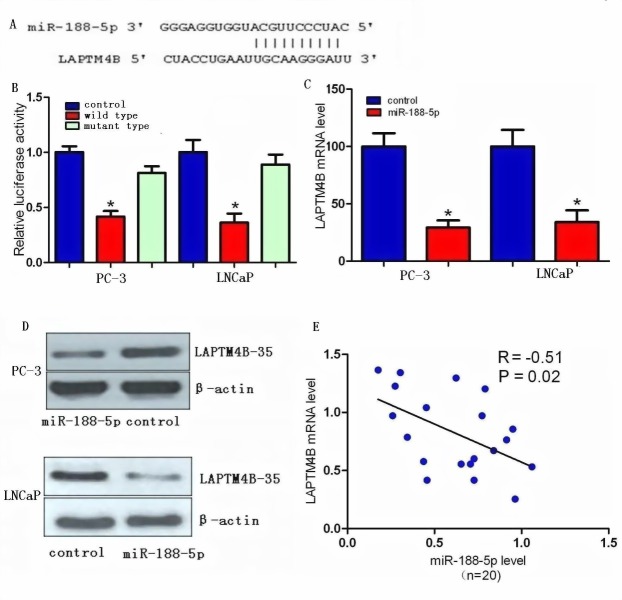
LAPTM4B is a direct target of miR-188-5p in PCa (A) Computational analysis showing that miR-188-5p potentially targeted LAPTM4B. (B) Relative luciferase activities were analysed in PC-3 and LNCaP cells. (C) Decrease in LAPTM4B mRNA expression by miR-188-5p was determined using qRT-PCR. (D) Decrease in LAPTM4B-35 protein expression by miR-188-5p was determined using western blot. β-actin was used as a loading control (E) LAPTM4B-35 mRNA expression was inversely correlated with miR-188-5p level in 20 pairs of PCa samples using linear regression models. All *P < 0.05.

We investigated the candidate targets for miR-188-5p using prediction algorithms provided by miRWalk, DIANA-mT, miRDB, TargetScan, PicTar5 and miRanda (www.umm.uni-heidelberg.de/apps/zmf/mirwalk/index.html). After integrating the results, we selected LAPTM4B for further validation due to its proliferation and metastasis properties in various types of cancers [9-16]. We next investigated whether LAPTM4B was a direct target of miR-188-5p or not. Through computational analysis, the binding site for miR-188-5p at 3′-UTR of LAPTM4B was depicted (Figure 1A). We then generated firefly luciferase reporter constructs with the 3′UTR of LAPTM4B mRNA, and transfected them into PC-3 and LNCaP cells with miR-188-5p mimics. We found that co-transfection with miR-188-5p in PC-3 and LNCaP cells decreased luciferase activity when the construct contained the 3′UTR of LAPTM4B (Figure 1B). Mutation of the binding sites reversed the observed inhibitory effects. These results suggested that LAPTM4B was a direct target of miR-188-5p.

We performed qRT-PCR and western blot in PC-3 and LNCaP cells to investigate whether restoration of miR-188-5p altered the expression of the LAPTM4B mRNA and protein. The mRNA and protein expression levels of LAPTM4B were significantly repressed in miR-188-5p transfectants as compared with control PC-3 and LNCaP cells (Figure 1C, D). We also find that the level of LAPTM4B mRNA was significantly upregulated in PCa tissues compared with paired normal prostate tissues from the same patients. Moreover, the upregulation of LAPTM4B mRNA was inversely correlated with the expression levels of miR-188-5p in 20 PCa tissue samples (Figure 1E). Collectively, our data showed that miR-188-5p negatively modulated LAPTM4B expression by directly binding to its 3′UTR.

### LAPTM4B as a potential metastasis-associated gene

**Figure 2:**
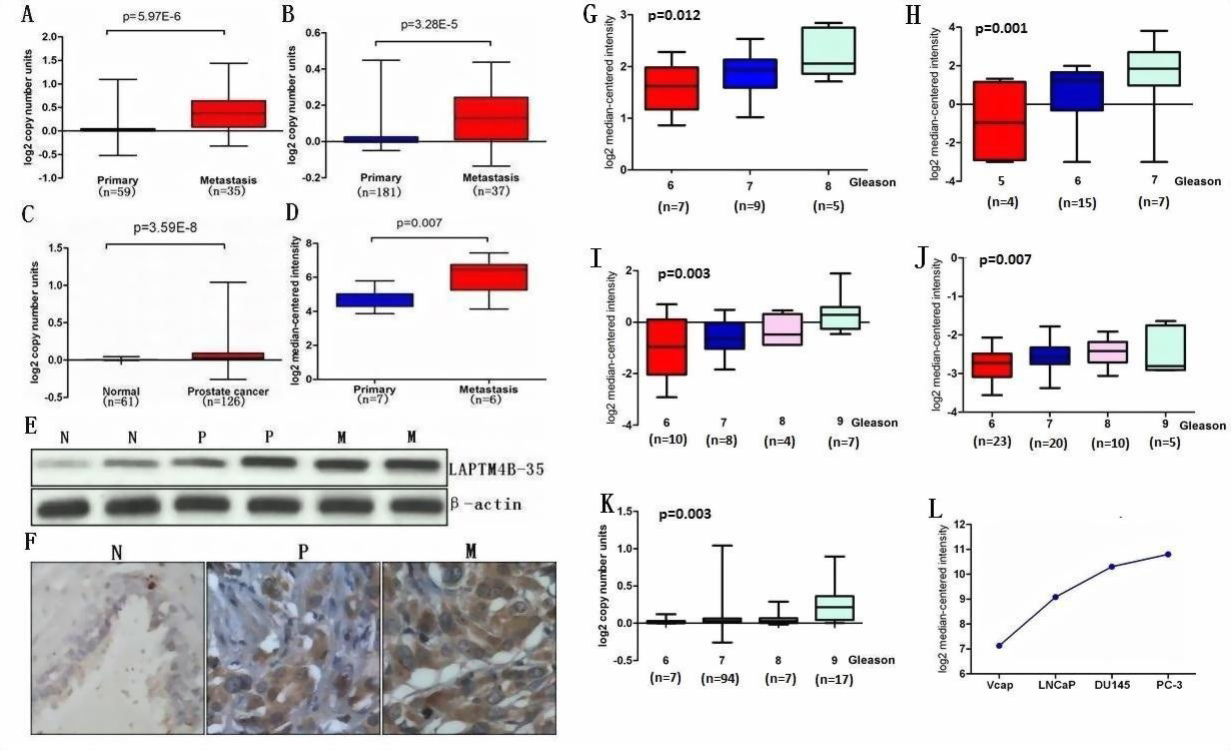
LAPTM4B is overexpressed in PCa and is associated with disease progression A, B, C and D, Box plots represent LAPTM4B copy number and mRNA level in normal prostate, primary PCa and metastatic PCa samples. The data was retrieved from available datasets of PCa with log2 copy number unites or log2 median intensity. E, LAPTM4B protein expression by immunoblot analysis of prostate tissue using LAPTM4B antibody. β-actin was used as a loading control. F, Immunohistochemical analysis of LAPTM4B in benign prostate epithelia (N), primary PCa (P) and metastatic PCa (M). G, H, I, J and K, LAPTM4B is significantly over-expressed in patients with higher Gleason score compared with patients with lower Gleason score. The data was retrieved from available microarray datasets of PCa with log2 copy number unites or log2 median intensity. L, LAPTM4B expression in PCa cell lines. The data was retrieved from available microarray dataset of PCa cell lines with log2 median intensity.

To assess whether any significant difference of LAPTM4B DNA copy number or mRNA level exists in metastatic PCa, primary PCa and normal prostate tissues, data from The Cancer Genome Atlas (TCGA) and some available datasets were analyzed [17-19]. Results showed that LAPTM4B DNA copy number was significantly increased in metastatic PCa samples compared with primary PCa samples (Figure 2A, B). Similarly, elevated LAPTM4B DNA copy number was observed in primary PCa compared with normal tissues (Figure 2C). Furthermore, expressions of LAPTM4B mRNA were higher in metastatic PCa samples than in primary PCa samples (Figure 2D).

In order to validate these observations, we performed qRT-PCR using RNA from metastatic PCa, primary PCa and normal prostate samples. qRT-PCR analysis confirmed the overexpression of LAPTM4B mRNA in metastatic PCa tissues relative to primary PCa and normal tissue as did immunoblot analysis using LAPTM4B-specific antibody (Figure 2E). We next investigated the expression of LAPTM4B protein in a group of PCa samples by immunohistochemical analysis that showed weak or no reactivity in benign tissues but strong staining in the aggressive PCa and metastatic PCa tissues (Figure 2F). We also conducted microarray data analyses and found that LAPTM4B is significantly over-expressed in patients with higher Gleason score compared with patients with lower Gleason score (Figure 2G-K) [17,20-23]. Consistent with the results from clinical samples, microarray analysis in PCa cell lines showed that Vcap and LNCaP expressed lower LAPTM4B levels in contrast to more aggressive PCa cell lines DU145 and PC-3 that express higher LAPTM4B levels (Figure 2L) [18]. Taken together, these results suggested LAPTM4B is overexpressed in PCa and is associated with disease progression.

### miR-188-5p suppresses cell proliferation, invasion and migration via the suppression of LAPTM4B

**Figure 3:**
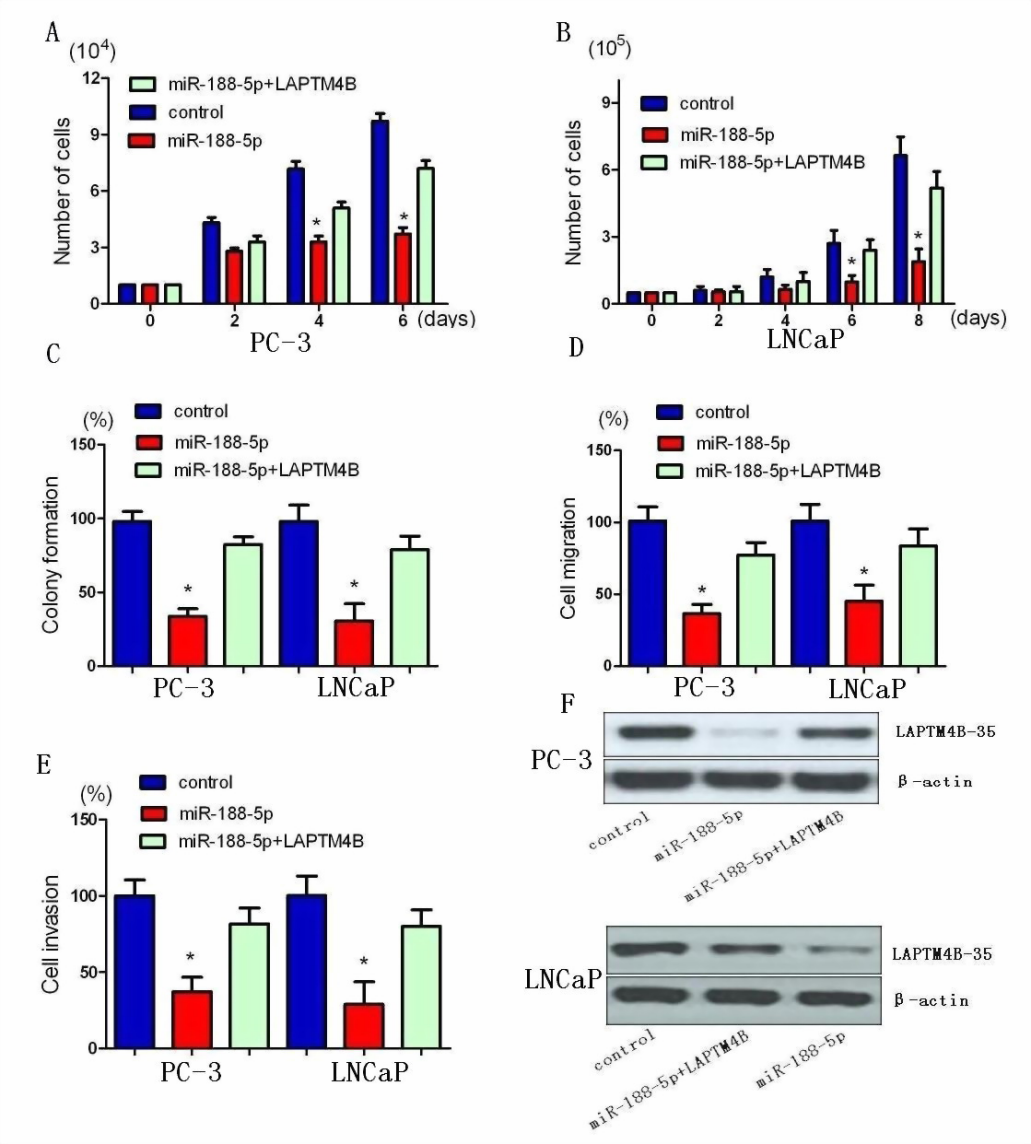
Restoration of LAPTM4B abrogates miR-188-5p-induced suppression of PCa growth, migration, and invasion A and B, Restoration of LAPTM4B significantly repressed the miR-188-5p-induced reduction of cell viability. C, Restoration of LAPTM4B significantly repressed the miR-188-5p-induced growth arrest in PCa by colony formation assay. D, Restoration of LAPTM4B reversed the inhibitory effects of miR-188-5p on cell migration. E, Restoration of LAPTM4B reversed the inhibitory effects of miR-188-5p on cell invasion. All data are shown as mean ± SD. F, Re-expression LAPTM4B in PC-3 and LNCaP cells was examined using western blotting analyses. All *P < 0.05.

We next confirmed whether LAPTM4B could affect the inhibitory effect of miR-188-5p on PCa progression. LAPTM4B was re-introduced in PC-3 and LNCaP cells. The results of the MTT assay showed that overexpression of LAPTM4B remarkly abrogated the suppression of PC-3 and LNCaP cell proliferation induced by miR-188-5p (Figure 3A, B). miR-188-5p-mediated loss of colony formation was also significantly antagonised by overexpression of LAPTM4B (Figure 3C). Furthermore, forced expression of LAPTM4B reversed the inhibition of PC-3 and LNCaP cell migration and invasion induced by miR-188-5p (Figure 3D, E).

The data showed that knockdown of LAPTM4B resulted in similar results induced by miR-188-5p expression in PC-3 and LNCaP cells. LAPTM4B expression was significantly decreased by miR-188-5p and LAPTM4B shRNA in PC-3 cells. As shown in MTT and colony formation assays, we found that both miR-188-5p and LAPTM4B knockdown induced a comparable inhibition of cell growth (Supplementary Figure 3A-C). When it comes to migration and invasion, LAPTM4B shRNA could mimic the inhibition of PC-3 and LNCaP cell migration and invasion induced by miR-188-5p (Supplementary Figure 3D, E). Taken together, these results indicated that miR-188-5p suppressed PC-3 and LNCaP cell proliferation, migration and invasion via the suppression of LAPTM4B.

### Restoration of miR-188-5p inhibits the PI3K/AKT signaling pathway via the suppression of LAPTM4B

**Figure 4:**
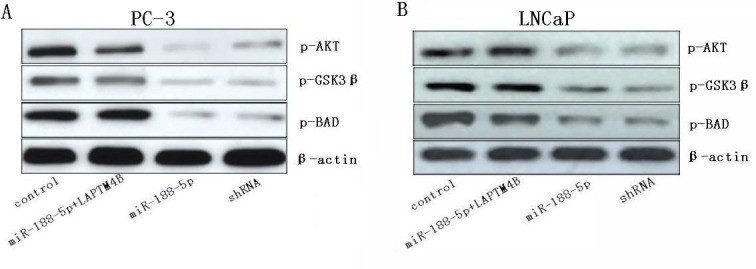
Restoration of miR-188-5p inhibits the PI3K/AKT signaling pathway via the suppression of LAPTM4B Levels of phosphorylations of AKT, GSK3β and Bad were determined in PC-3 and LNCaP cells transfected with miR-188-5p and/or LAPTM4B.

Previous study demonstrated that upregulation of LAPTM4B-35 in HeLa cells can markedly activate the PI3K/AKT signaling pathway [24]. The phosphorylation of AKT was an indication of PI3K/AKT signaling activation. GSK3β and Bad were the downstream substrates of AKT and can be phosphorylated by activated AKT. Western blot analysis showed that phosphorylations of AKT, GSK3β and Bad were decreased in miR-188-5p overexpression cells compared with control cells (Figure 4). The alterations of these proteins involved in PI3K/AKT signaling pathway can be rescued by LAPTM4B restoration. Taken together, these results indicated that restoration of miR-188-5p suppressed PI3K/AKT signaling pathway by targeting LAPTM4B.

### Restoration of miR-188-5p promotes PC-3 cells' sensitivity to adriamycin via the suppression of LAPTM4B

**Figure 5:**
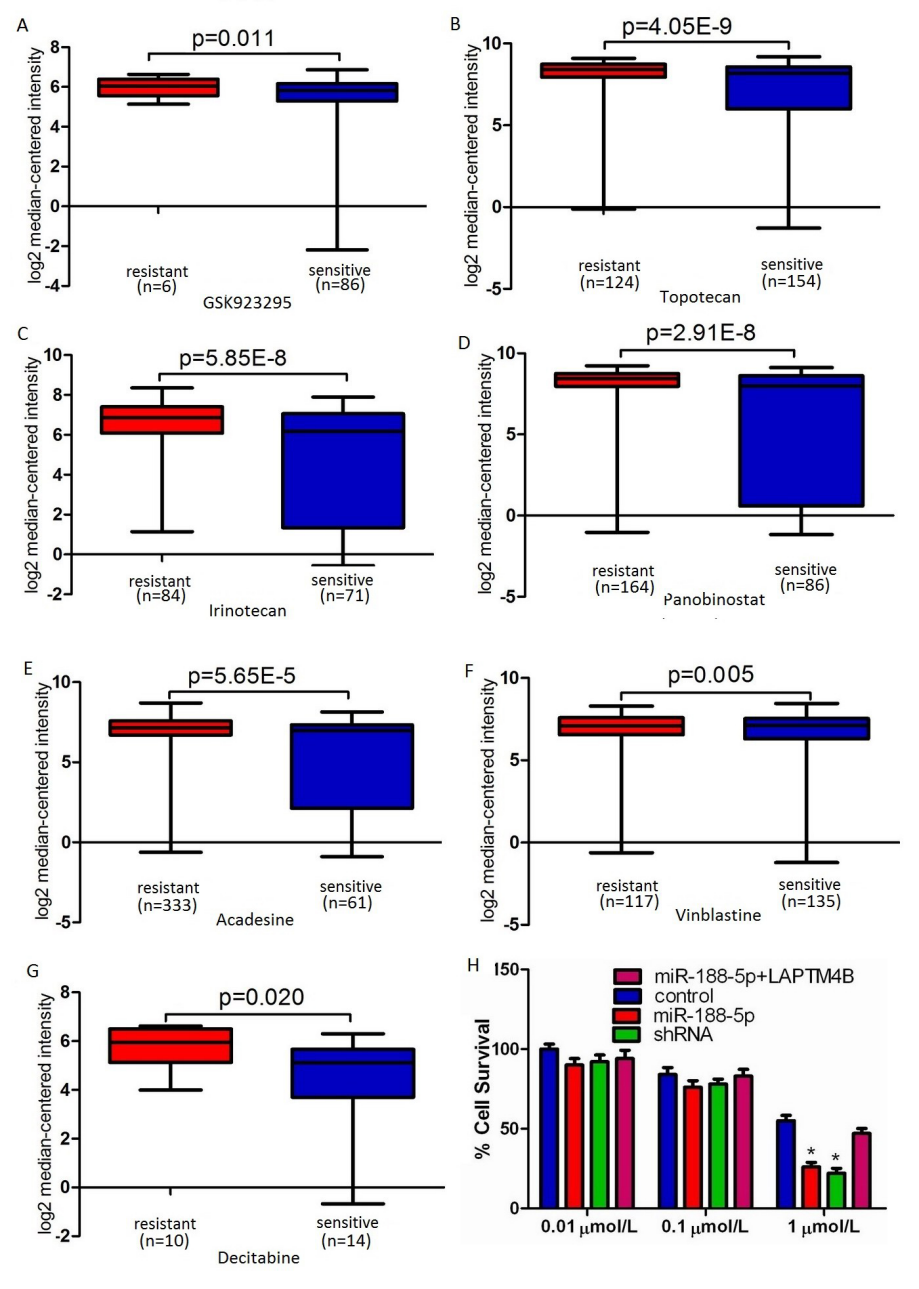
Restoration of miR-188-5p promoted PC-3 cells' sensitivity to adriamycin via the suppression of LAPTM4B A-G, LAPTM4B expression was up-regulated in drug resistant cancer cell lines compared to drug sensitive cancer cell lines. The data were retrieved from available datasets with a log2 fold change. H, miR-188-5p overexpression can significantly enhance the chemosensitivity of PC-3 cells to adriamycin (1μmol/L), compared with the control groups. Forced overexpression of LAPTM4B reversed the promotion of the chemosensitivity of PC-3 cells to adriamycin induced by miR-188-5p. All data are shown as mean ± SD. All *P < 0.05.

LAPTM4B motivates multidrug resistance by activation of PI3K/AKT signaling pathway to escape from drug-induced apoptosis [15]. We performed microarray data analyses and revealed that LAPTM4B is significantly over-expressed in multidrug resistant cancer cell lines compared with drug sensitive cancer cell lines (Figure 5A-G) [25-27]. Therefore, LAPTM4B silencing may improve the cancer cells' sensitivity to chemotherapeutic drugs. To investigate whether miR-188-5p overexpression can change the PC-3 cells' sensitivity to chemotherapeutic drugs, the inhibition values for adriamycin in these cells were determined by MTT assay. As in Figure 5H, the statistical analysis showed that the miR-188-5p overexpression could significantly enhance the chemosensitivity of PC-3 cells to adriamycin (1μmol/L), compared with the control groups. Forced overexpression of LAPTM4B reversed the promotion of the chemosensitivity of PC-3 cells to adriamycin induced by miR-188-5p (Figure 5H). We also found that both miR-188-5p overexpression and LAPTM4B knockdown could result in a comparable promotion of the chemosensitivity of PC-3 cells to adriamycin (Figure 5H). Collectively, these data showed that restoration of miR-188-5p promoted PC-3 cells' sensitivity to adriamycin via the suppression of LAPTM4B.

### Ectopic expression of miR-188-5p inhibits tumour growth and metastasis *in vivo*

**Figure 6:**
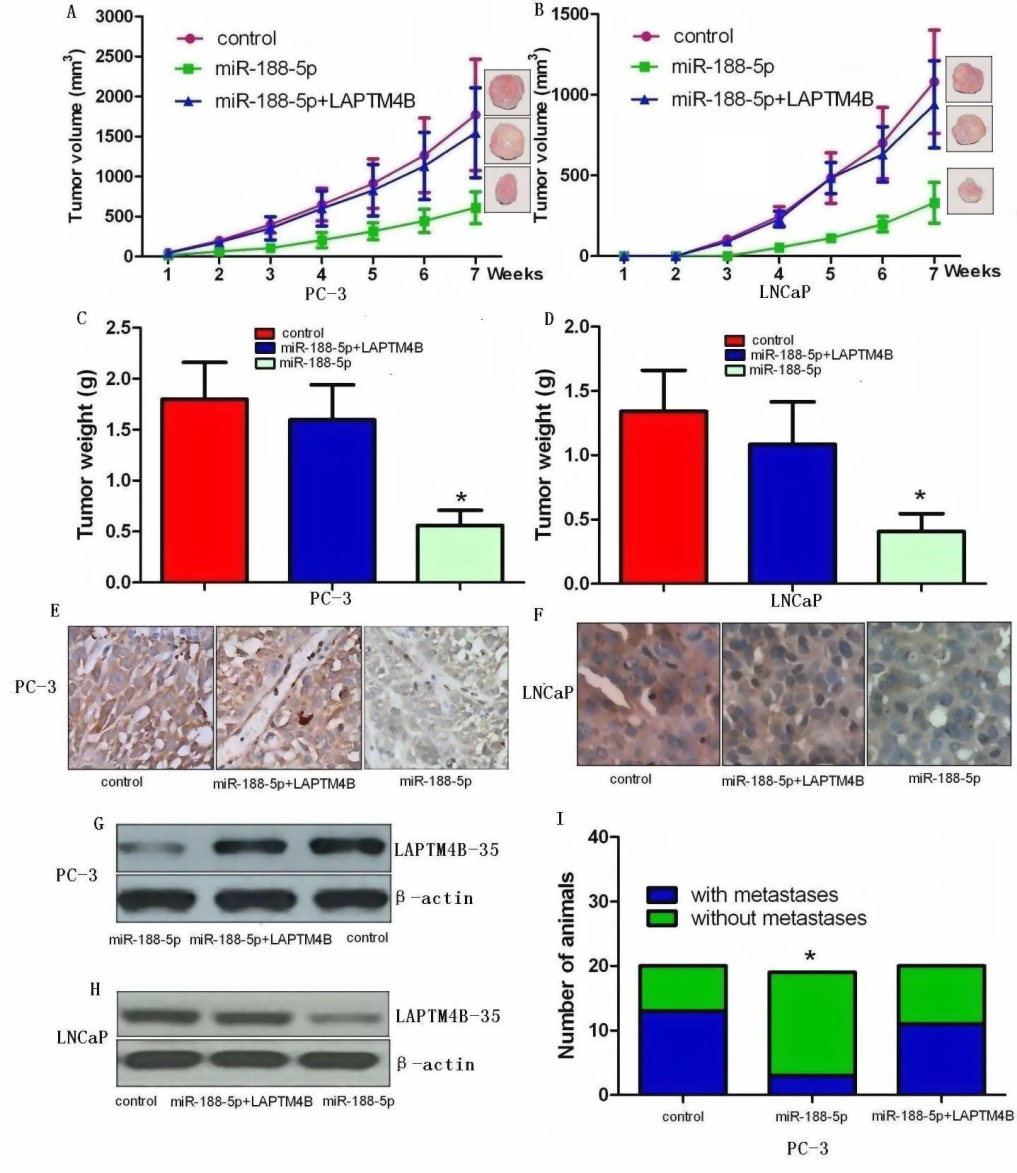
Ectopic expression of miR-188-5p inhibits tumour growth and metastasis via targeting LAPTM4B *in vivo* Restoration of LAPTM4B significantly reversed the suppression of tumour growth and metastasis induced by miR-188-5p. A and B, Ectopic expression of miR-188-5p in PC-3 and LNCaP cells significantly inhibits tumour growth in a mouse xenograft model. All data are shown as mean ± SD. C and D, Tumour weights of corresponding mouse xenograft models. All bar graphs are shown with ± SEM. E and F, LAPTM4B expression analysis was performed at protein level by immunochemistry (original magnification X 400). G and H, LAPTM4B expression analysis was conducted at protein level by western blot. I, miR-188-5p played an important role in PCa metastasis *in vivo* via targeting LAPTM4B. All *P < 0.05.

In order to assess the role of miR-188-5p on tumour growth *in vivo*, we examined miR-188-5p-mediated tumorigenesis in a murine xenograft model using control, miR-188-5p+LAPTM4B PC-3 and LNCaP cells, and PC-3 and LNCaP cells expressing miR-188-5p. Stable PC-3 and LNCaP cells expressing miR-188-5p significantly reduced tumour growth and weight in mice (Figure 6A-D) relative to control xenograft models demonstrating that restoration of miR-188-5p attenuates tumour growth *in vivo*. On histological sections, xenograft tumours expressing miR-188-5p showed decreased staining for LAPTM4B-35 (Figure 6E-H). To assess the role of LAPTM4B on tumour metastasis *in vivo*, we measured metastases exclusively in the cervical lymph nodes. Analyses displayed attenuated metastasis in the miR-188-5p expressing PC-3 group compared to the control group (Figure 6I). Consistent with this *in vitro* data, restoration of LAPTM4B significantly reversed the suppression of tumour growth and metastasis induced by miR-188-5p. We did not find any metastases in the cervical lymph nodes in control or experimental LNCaP xenografts. These observations clearly showed that downregulation of miR-188-5p played an important role in PCa growth and metastasis *in vivo* via targeting LAPTM4B.

## DISCUSSION

Aberrantly expressed miRNAs can disrupt the tightly regulated RNA networks in cancer cells, triggering cancer development and metastasis [28,29]. To date, there has been no report about miR-188-5p functional analyses in PCa. This is the first report that aimed to investigate the functional significance of miR-188-5p in PCa. Here, we reported that miR-188-5p was down-expressed in PCa and that downexpression of miR-188-5p might serve as an independent predictor for the poor outcome of PCa patients. The data suggested that downexpression of miR-188-5p was significantly correlated with metastases. We found that miR-188-5p inhibited PCa cell proliferation, invasion and migration through the downregulation of LAPTM4B by directly binding to its 3′-UTR. We further found that miR-188-5p can regulate cell response under chemotherapy. Taken together, these results demonstrated that miR-188-5p played an important role in PCa progression and chemotherapeutic drug sensitivity.

PSA is the most common biomarker for PCa detection and for following the course of metastatic PCa [30,31]. However, the course of PCa progression and clinical outcomes of PCa patients can differ even in patients with the same PSA level, Gleason score and pathological stage. Therefore, it is crucial to identify more sensitive markers for improvement of PCa prognosis. To investigate this, we analyzed the expression level of miR-188-5p and its association with the clinicopathological features of PCa patients. We also performed univariate and multivariate logistic regressions to assess the association between miR-188-5p downexpression and lymph node metastasis. The results suggested that downregulation of miR-188-5p was an important molecular biomarker in identifying poor outcomes for PCa patients. The results also suggested that downregulation of miR-188-5p was independently associated with lymph node metastases. Taken together, downexpression of miR-188-5p was associated with metastasis and an independent prognostic biomarker for worse survival. A further large-scale cohort study may be necessary to determine whether miR-188-5p is an effective marker.

In current study, miR-188-5p was identified to be down-regulated in PCa, and furthermore, its decreased expression is associated with poor prognosis in patients with PCa, which strongly suggests a potential role of miR-188-5p in suppression of PCa. We conducted miRNA microarray data analyses and found that miR-188-5p may be a potential metastasis-associated miRNA. These results indicated that the downexpression of miR-188-5p in PCa may facilitate the invasive/metastatic phenotype. However, its underlying mechanism is not well-understood. To further understand the mechanism by which miR-188-5p exhibits tumor suppressive function in PCa, we performed a computational search for the potential targets for miR-188-5p. LAPTM4B was identified as a potential target for miR-188-5p. LAPTM4B located in chromosome 8q22.1 and encoded a 35-kDa protein, LAPTM4B-35, which was a type-III transmembrane protein with four putative transmembrane regions. Mounting evidences show that overexpression of LAPTM4B-35 is associated with a poor prognosis and contributes to cellular transformation, tumorgenesis, and metastatic progression in several human cancers [9-14]. Our data showed that the overexpression of LAPTM4B mRNA and protein in metastatic PCa tissues relative to primary PCa and normal tissue. We conducted microarray data analyses and found that LAPTM4B is significantly over-expressed in patients with higher Gleason score compared with patients with lower Gleason score. We also found that Vcap and LNCaP expressed lower LAPTM4B levels in contrast to more aggressive PCa cell lines DU145 and PC-3. These results suggested that the overexpression of LAPTM4B is associated with PCa progression. In PCa samples, the expression level of miR-188-5p was found to be correlated inversely with LAPTM4B mRNA expression. We found that miR-188-5p can negatively regulated LAPTM4B expression in PCa. We further characterised LAPTM4B as a direct target of miR-188-5p.

Proliferation and metastasis, two hallmarks of malignancy, are the leading causes for the cancer-related death [32,33]. Ectopic miR-188-5p expression significantly repressed the proliferation, migration and invasion of PC-3 and LNCaP cells *in vitro*, and tumour growth and metastasis *in vivo*. These results demonstrated that PC-3 and LNCaP cells became less aggressive and invasive after transfected with the miR-188-5p expression construct, suggesting that the miR-188-5p functioned as a tumour suppressor in PCa. Our data further demonstrated that the tumour-suppressive miR-188-5p targets LAPTM4B and that silencing of LAPTM4B significantly inhibits the proliferation, migration and invasion of PC-3 and LNCaP cells. In addition, forced expression of LAPTM4B can significantly reverse the suppressive effects of miR-188-5p. Collectively, our study provides experimental evidence that miR-188-5p suppresses cell growth and metastasis in PCa by directly targeting LAPTM4B.

The abilities of survival and metastasis to large distance are often complied with tumours that are resistant to chemotherapeutic drug treatment. Patient response to chemotherapy has shown to be closely correlated to the functional status of miRNAs [34-36]. Although the molecular mechanism of miRNA-regulated drug resistance still remains largely unknown, the data suggest several functional roles for miRNAs, including influence of therapeutic induced cell death, regulation of multiple drug resistance related proteins, change in bioavailable drug concentration, alteration of drug targets and promotion of angiogenesis and tumour stem-like cells [37]. More and more experiments have recently revealed that the activation of PI3K/AKT signaling pathway can regulate or enhance multidrug resistance [15,38-41]. Cancer cells can activate PI3K/AKT signaling pathway, which modulate cell survival signaling and regulate DNA repair machinery directly [42].

Our data showed that phosphorylations of AKT, GSK3β, and Bad were decreased in miR-188-5p overexpression cells. The alterations of these proteins involved in PI3K/AKT signaling pathway can be rescued by LAPTM4B restoration. Our data also showed that restoration of miR-188-5p can promote PC-3 cells' sensitivity to adriamycin via the suppression of LAPTM4B. Previous study demonstrated that LAPTM4B can motivate multidrug resistance of cancer cells by promoting drug efflux and anti-apoptosis by activating PI3K/AKT signaling [15,16,32]. Collectively, these results indicated that restoration of miR-188-5p suppressed PI3K/AKT signaling pathway by targeting LAPTM4B. All the data indicated that the intracellular miR-188-5p may modulate sensitivity of cancer cells to adriamycin through targeting LAPTM4B with important implications in the design of new therapeutic agents.

Several potential limitations of the present study should be taken into consideration. A correction for multiple testing has not been done in the present study. Thus, the results should be interpreted cautiously. The sample sizes in some of the experiments were small. Further large-scale analyses will be needed in the future. Although the newly identified miR-188-5p/LAPTM4B/PI3K/AKT signaling pathway offers new insights into the pathogenesis of PCa and a potential therapeutic target for PCa treatment, the molecular mechanism silencing the expression of the miR-188-5p in PCa is still unknown, analysis of the detailed molecular mechanism will be necessary in future studies.

In this line of research, this is the first report unveiled that miR-188-5p as a novel player with tumour suppressor functions in the cancerogenesis of PCa. Furthermore, miR-188-5p/LAPTM4B/PI3K/AKT regulatory network played an important role in PCa progression and chemotherapeutic drug sensitivity. Elucidation of the molecular mechanism should lead to the development of more effective therapeutic strategies for PCa patients in the future.

## MATERIALS AND METHODS

### Patients

A total of 180 human PCa and paired adjacent noncancerous tissues were obtained from the second hospital of Tianjin medical university, which underwent radical prostatectomy at this hospital between 1999 and 2010 [43,44]. None of the patients had received androgen deprivation treatment, chemotherapy or radiation therapy prior to the surgery. We define biochemical failure as a PSA level exceeding 0.2 ng/mL on at least two successive evaluations after radical prostatectomy. Biochemical recurrence free survival: the period between surgical treatment and the measurement of two successive values of serum PSA level ≥ 0.2 ng/ml. This study was approved by the research ethics committee of Tianjin medical university. This investigation conformed to the principles outlined in the Declaration of Helsinki.

### Cell Lines

PCa cell lines PC-3 and LNCaP were grown in RPMI 1640 (Life Technologies, CA) with 0.023 IU/ml insulin and 10% FBS (Invitrogen) in 5% CO_2_ cell culture incubator.

### Plasmids and Cell transfection

The pSilencer-shRNA plasmids for knockdown of LAPTM4B were constructed with pSilencer™ hygro vectors (Ambion, Austin, TX) according to the manufacture's instructions. The target sequence of LAPTM4B shRNA was ATGCTGTGGTACTGTTGATTT [45]. The expression vector pcDNA3.1 containing LAPTM4B was constructed according to the manufacture's instructions, which was used for “rescue” experiments. miR-188-5p mimics were purchased from Ambion (Sigma-Aldrich, Saint Louis, MO, USA). For transient transfection of cells in 6-well plates, 200 μM mimics were used with Lipofectamine 2000 (Invitrogen). To construct the miR-188-5p expressing vector, pri-miR-188-5p was amplified and cloned into pcDNA3.1 (Invitrogen). PC-3 and LNCaP cells were plated in 6-well plates for stable transfection with 400mg pcDNA3.1-miR-188-5p using Effectene (Qiagen). All plasmid constructions were verified by nucleotide sequence analysis

### Proliferation assays

Cell viability/proliferation was measured using the MTT assay. Cells in the logarithmic growth phase were harvested and seeded on 96-well plates. MTT assay was performed by adding 20 ul of MTT (Sigma-Aldrich, Saint Louis, MO, USA) for 4 hour at 37°C. 200 μl of DMSO was added to each well at the end of the culturing period and the plates were agitated for 15 minutes. The absorbance at 490 nm of each sample was measured using a multilabel plate reader (Bio-Rad 3350). In colony formation assay, the cells were seeded on 35-mm dishes. The cells were fixed in methanol, and then stained with crystal. Finally, positive colony formation (>50 cells/colony) was counted.

### Cell migration and invasion assay

Cells were counted and plated equally in the upper compartments of the 48-well bodyben chamber, 8-mm-pore-size polycarbonate membrane filters. Cell migration and invasion assays were performed with uncoated (migration) and coated Matrigel (invasion). After 24 hours, the migrated and invaded cells in the membrane were fixed with methanol and stained with crystal violet, and the cells were counted under a microscope. All assays were performed in triplicate.

### Luciferase reporter assay

In brief, the miR-188-5p-binding site in the LAPTM4B 3′-UTR region (wild or mutant-type) was cloned downstream of the firefly luciferase gene in a pGL3-promoter vector (Promega, Madison, WI). For the luciferase assay, cells were cultured in 12-well plates and contransfected with 50 nM of oligonucleotide, 200 ng of the luciferase reporter construct and 20 ng of the renilla luciferase reporter construct. After 48 hours, luciferase activity was measured using the dual luciferase reporter assay system (Promega, Madison, WI).

### RNA extraction and qRT-PCR analyses

Total RNA was extracted with TRIzol reagents (Invitrogen, Carlsbad, CA). qRT-PCR was performed to quantify miRNA expression with the NCode miRNA qRT-PCR analysis (Invitrogen, Carlsbad, CA), or mRNA expression with the Takara SYBR Premix Ex Taq II (Takara, Japan). qRT-PCR was performed with the Applied Biosystems 7300 real-time PCR system (Applied Biosystems). The primer for miR-188-5p was 5′-CATCCCTTGCATGGTGGAGGG-3′. β-actin or control miRNA U6 was used for normalization. The primers used for mRNA expression were obtained from the PrimerBank database (http://pga.mgh.harvard.edu/primerbank/).

### Western blot analysis

Total protein from the PCa cells was extracted with RIPA lysis buffer. Protein concentration was measured by use of the BCA reagent kit (Merck). The protein was resolved by SDS-PAGE and transferred to a PVDF membrane, which was probed with specific primary antibodies against LAPTM4B-35 (LAPTM4BN199-pAb; Abcam Co) and phosphorylations of AKT (Ser473), GSK3β (Ser9), and Bad (Ser136). β-actin was blotted to show equal protein loading.

### PC-3 cells' chemosensitivity to adriamycin studies

Adriamycin was initially dissolved in 0.9% sodium chloride and was diluted with fresh medium immediately before each experiment. The concentration of adriamycin varied from 0.01 to 1μmol/L. The PC-3 cell survival was determined using MTT assay. 20μl MTT was added to each well and incubated at 37°C for 4 hours. Then the medium in each well was removed and 150μl DMSO was added. The number of PC-3 cells was assessed by measurement of absorbance at 492 nm using Multiskan MS.

### Prostate tumour xenograft studies

We established xenografts in nude mice with the stable expressing miR-188-5p cells, LAPTM4B+miR-188-5p (LAPTM4B plus miR-188-5p) cells, and control cells. 1 × 10^6^ (PC-3) or 6 × 10^6^ (LNCaP) cells were implanted into the dorsal flank of male Athymic nude mice (n = 20 per group, 5 weeks of age) subcutaneously. Tumour size was measured biweekly, and tumour volumes were calculated using the formula: Volume (mm^3^) = [width^2^ (mm^2^) × length (mm)]/2. Mice with tumours were killed 7 weeks after the inoculation. The xenograft tumours, and the cervical lymph nodes were collected and tumour weights were measured. DNA extraction of the cervical lymph nodes and human alu sequence PCR amplification were performed as described previously [46]. All experiments involving animals were conducted according to the Animal Welfare Act and approved by Animal Care and Use Committee of the Tianjin Medical University (Approval number TMUaMEC2013021).

### Statistical analysis

For continuous variables, Student's t-test was performed. Spearman correlation test was chosen for examining the correlations between miR-188-5p expression level and the clinical and pathological variables. Univariate and multivariate logistic regressions with covariate adjustment were used to assess the association between miR-188-5p downexpression and pelvic lymph node metastasis. Survival curves were carried out by the Kaplan-Meier method and evaluated using the log-rank test. Identified factors were associated with survival by the Cox proportional hazard regression model. P<0.05 was considered statistically significant. Statistical analysis was performed using SPSS 17.0 software.

## SUPPLEMENTARY MATERIAL, FIGURES, TABLES


