# Supplementary material for: miR-188-5p inhibits tumour growth and metastasis in prostate cancer by repressing LAPTM4B expression
Source: Oncotarget. 2015 Jan 21;6(8):6092–104. doi: 10.18632/oncotarget.3341 (PMC4467424; doi:10.18632/oncotarget.3341)
Supplement: Supplementary file 1 [file oncotarget-06-6092-s001.pdf]

miR-188-5p inhibits tumour growth and metastasis in prostate cancer by repressing LAPTM4B expression

Supplementary Material

Supplementary Table 1: Clinicopathologic variables and miR-188-5p expression in 180 PCa patients. We define biochemical failure as a PSA level exceeding 0.2 ng/mL on at least two successive evaluations after radical prostatectomy.

Variable	Group	miR-188-5p expression			P value
		High (n=90)	Low (n=90)	Total	
Age	＜70	49	48	97	0.881
	≥70	41	42	83	
Lymph node metastasis	Absence	86	77	163	0.022
	Presence	4	13	17	
Surgical margin status	Absence	85	81	166	0.266
	Presence	5	9	14	
Seminal vesicle invasion	Absence	81	64	145	0.001
	Presence	9	26	35	
Clinical stage	T1	60	43	103	0.010
	T2/T3	30	47	77	
Preoperative PSA	＜4	4	1	5	0.024
	4-10	39	25	64	
	＞10	47	64	111	
Gleason score	＜7	68	31	99	＜0.001
	7	14	20	34	
	＞7	8	39	47	
Angiolymphatic invasion	Absence	82	73	145	0.001
	Presence	8	27	35	
Biochemical recurrence	Absence	71	57	128	0.021
	Presence	19	33	52	

Supplementary Table 2: Factors predictive of lymph node metastasis in the univariable and multivariable logistic regressions.

Variable	B	S.E.	OR	95% CI	P value
**Univariable analysis**					
Prostate cancer Stage	0.199	0.311	1.220	0.663-2.234	0.523
Gleason score	0.068	0.216	1.070	0.701-1.633	0.753
Preoperative prostate-specific antigen	0.737	0.320	2.089	1.116-3.911	0.021
Age	0.224	0.215	1.251	0.820-1.908	0.299
Surgical margin status	-0.265	0.336	0.767	0.397-1.483	0.430
Presence of seminal vesicle invasion	0.166	0.216	1.181	0.773-1.805	0.442
miR-188-5p downexpression	1.078	0.258	2.938	1.773-4.870	<0.001
Presence of angiolymphatic invasion	0.547	0.218	1.728	1.128-2.648	0.012
**Multivariable analysis**					
Preoperative prostate-specific antigen	0.248	0.348	1.282	0.647-2.583	0.478
miR-188-5p downexpression	0.983	0.274	2.671	1.562-4.568	<0.001
Presence of angiolymphatic invasion	0.479	0.224	1.615	1.040-2.507	0.033

Supplementary Table 3: Prognostic value of miR-188-5p expression for the biochemical recurrence free survival in univariate and multivariate analyses by Cox regression. Biochemical recurrence free survival: the period between surgical treatment and the measurement of two successive values of serum PSA level ≥ 0.2 ng/ml.

Covariant	Univariate analysis			Multivariate analysis		
	Exp (B)	95% CI	P value	Exp (B)	95% CI	P value
miR-188-5p expression	2.210	1.403-3.484	0.001	2.111	1.338-3.330	0.001
Gleason score	1.703	1.280-2.265	<0.001	1.676	1.261-2.229	<0.001
Seminal vesicle invasion	1.505	1.132-2.003	0.005	1.446	1.087-1.922	0.011
Preoperative PSA	1.241	0.705-2.188	0.454			
Angiolymphatic invasion	1.084	0.814-1.443	0.580			
Surgical margin status	1.017	0.709-1.459	0.925			
PCa Stage	1.090	0.921-1.291	0.316			
Lymph node metastasis	1.140	0.850-1.528	0.381			
Age	1.068	0.804-1.419	0.650			

Supplementary Table 4: Prognostic value of miR-188-5p expression for the overall survival in univariate and multivariate analyses by Cox regression.

Covariant	Univariate analysis			Multivariate analysis		
	Exp (B)	95% CI	P value	Exp (B)	95% CI	P value
miR-188-5p expression	4.096	2.394-7.008	<0.001	3.007	1.736-5.206	<0.001
Gleason score	2.526	1.788-3.568	<0.001	1.889	1.320-2.702	<0.001
Preoperative PSA	2.034	1.338-23.092	0.001	2.277	1.481-3.502	<0.001
PCa Stage	4.131	2.888-5.911	<0.001	3.017	2.057-4.426	<0.001
Age	1.282	0.917-1.792	0.146			
Angiolymphatic invasion	1.373	0.813-2.319	0.235			
Surgical margin status	1.101	0.703-1.724	0.674			
Lymph node metastasis	1.044	0.746-1.462	0.800			
Seminal vesicle invasion	1.358	0.956-1.928	0.087			

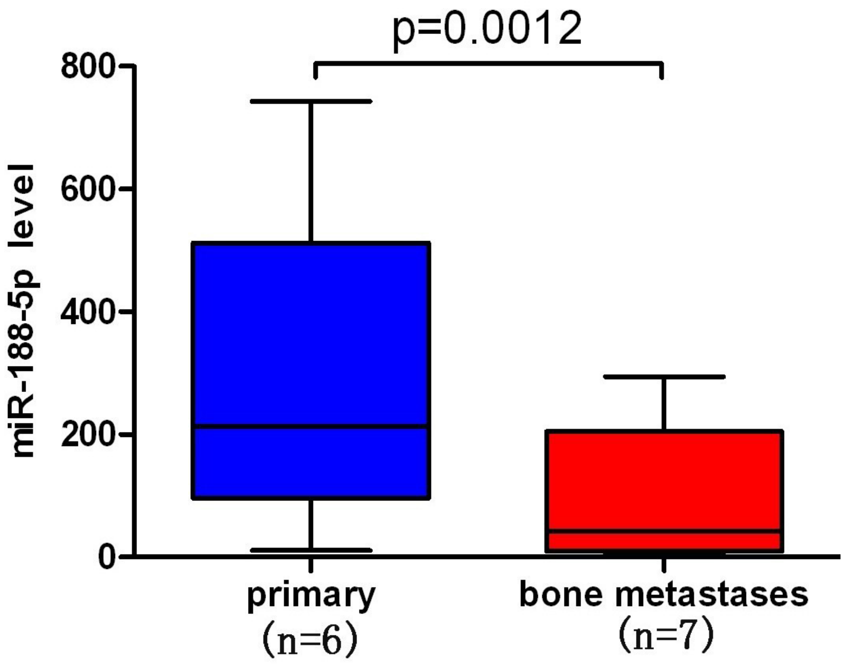

Figure S1: miR-188-5p is under-expressed in metastasis PCa. miR-188-5p expression was down-regulated in metastatic PCa compared to primary PCa. Expression was shown as a log2 fold change.

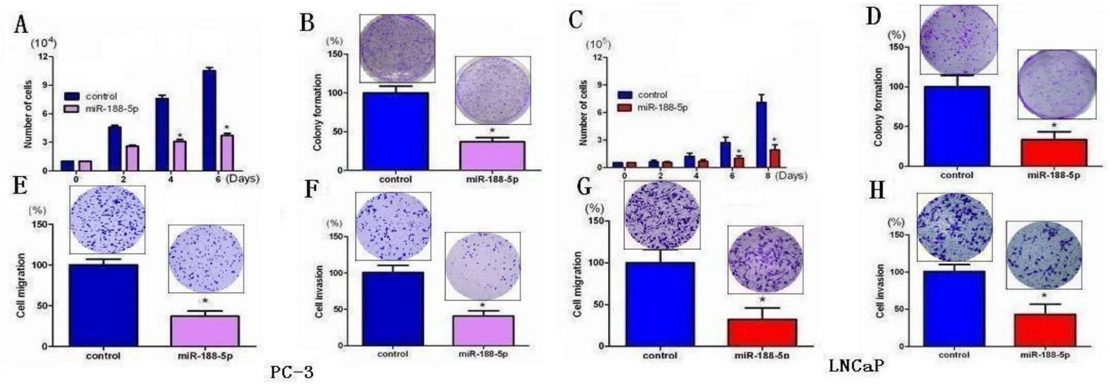

Figure S2: miR-188-5p inhibits PC-3 and LNCaP cell proliferation, migration and invasion *in vitro*. A, miR-188-5p reduced PC-3 cell proliferation. Cell viabilities were determined using MTT assays. B, miR-188-5p suppressed PC-3 colony formation. C, miR-188-5p reduced LNCaP cell proliferation. Cell viabilities were determined using MTT assays. D, miR-188-5p suppressed LNCaP colony formation. E, PC-3 Cell migration activity after transfection with miR-188-5p was determined using migration assays. F, PC-3 Cell invasion activity after transfection with miR-188-5p was determined using Matrigel invasion assays. G, LNCaP Cell migration activity after transfection with miR-188-5p was determined using migration assays. H, LNCaP Cell invasion activity after transfection with miR-188-5p was determined using Matrigel invasion assays. All data are shown as mean ± SD. All $*P < 0.05$.

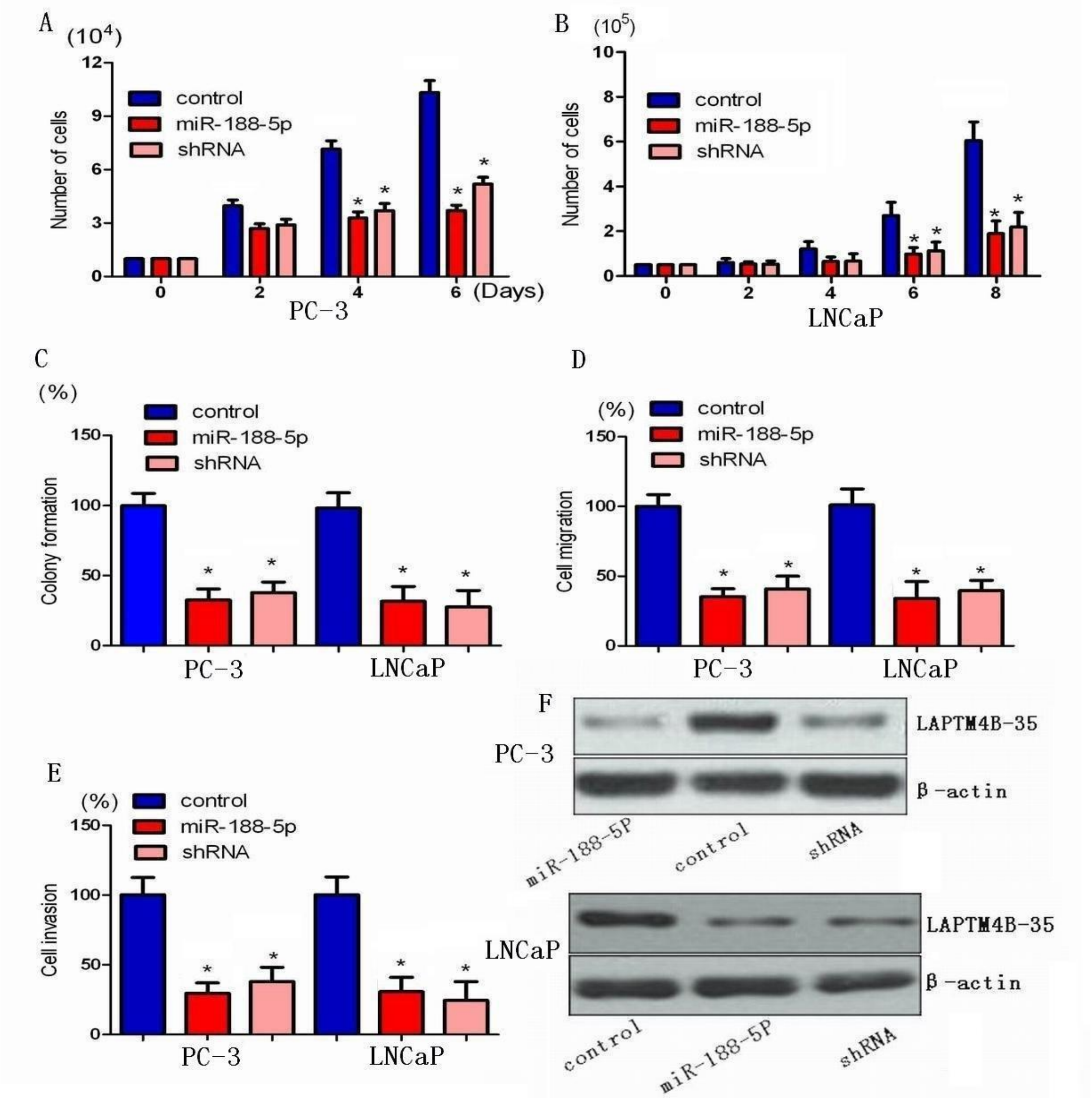

Figure S3: Knockdown of LAPTM4B mimics the effects of miR-188-5p on PC-3 and LNCaP cells. A and B, Knockdown of LAPTM4B can mimic the suppression of cell viability induced by miR-188-5p in PC-3 and LNCaP cells. C, Knockdown of LAPTM4B can mimic the growth arrest induced by miR-188-5p by colony formation assay. D, Knockdown of LAPTM4B can mimic the inhibitory effects of miR-188-5p on cell migration. E, Knockdown of LAPTM4B can mimic the inhibitory effects of miR-188-5p on cell invasion. F, The efficiency of LAPTM4B gene silencing was confirmed at protein level by western blot. All *$P < 0.05$.
